# *Microsporum canis* Causes Cutaneous and Extracutaneous Feline Dermatophytic Pseudomycetomas: Molecular Identification and Clinicopathological Characteristics

**DOI:** 10.3390/jof10080576

**Published:** 2024-08-14

**Authors:** Stefan Hobi, Wing Yan Jacqueline Tam, May Tse, Omid Nekouei, Yingfei Chai, Fraser I. Hill, Edmund Cheung, Wietz Botes, Francois Saulnier-Troff, Colin T. McDermott, Vanessa R. Barrs

**Affiliations:** 1 Department of Veterinary Clinical Sciences, Jockey Club College of Veterinary Medicine & Life Sciences, City University of Hong Kong, Kowloon Tong, Hong Kong, Chinajacqutam4@cityu.edu.hk (W.Y.J.T.); maypy.tse@cityu.edu.hk (M.T.); yingchai_chai@126.com (Y.C.); c.mcdermott@cityu.edu.hk (C.T.M.); 2 City University Veterinary Medical Centre, Hong Kong, China; francois.saulnier@vsh.com.hk; 3 Veterinary Diagnostic Laboratory, City University of Hong Kong, Kowloon Tong, Hong Kong, China; fraser.hill@cityuvdl.com.hk; 4 Department of Infectious Diseases and Public Health, Jockey Club College of Veterinary Medicine & Life Sciences, City University of Hong Kong, Kowloon Tong, Hong Kong, China; omid.nekouei@cityu.edu.hk; 5 Not for Profit Veterinary Clinic, Prince Edward, Hong Kong, China; ec200100@gmail.com; 6 Family Vet Fo Tan, Shatin, Hong Kong, China; wietzb@yahoo.co.uk; 7 Centre for Animal Health and Welfare, City University of Hong Kong, Kowloon Tong, Hong Kong, China

**Keywords:** feline, invasive fungal infection, moulds, dermatophytes, pseudomycetoma, eumycetoma

## Abstract

Dermatophytic pseudomycetoma (DPM) is a rarely reported invasive fungal infection of humans and animals, especially cats. This study aimed to identify dermatophytes, breed associations, and the frequency of extracutaneous (EC) involvement in feline DPM. Electronic records and formalin-fixed paraffin-embedded tissue (FFPET) from 32 suspected DPM cases in 30 cats were retrieved from a diagnostic laboratory between 2018 and 2024. To confirm DPM and molecular identity, DNA was extracted from FFPET for ITS2 sequencing, and immunohistochemistry was performed on PCR-negative cases. All cases were confirmed as DPM. *Microsporum canis* was the only dermatophyte identified. The sensitivity and specificity of ITS2 sequencing for *M. canis* identification in FFPET were 22/32 (68.8%) and 21/22 (95.5%), respectively. Exotic (36.7%) and Persian (23.3%) but not British breeds (26.3%) were over-represented compared to feline admissions at an affiliated veterinary hospital (8.5%, *p* < 0.001; 3%, *p* < 0.001; 21.6%, *p* = 0.817, respectively). Five cases (16.7%) had EC lesions; two had intra-abdominal masses; two had oral cavity masses, including one which extended into the cranial vault; and one had superficial cervical lymph node invasion. Exotic and Persian breeds are over-represented for DPM and *M. canis* is the primary cause. EC lesions of DPM may occur more commonly than previously thought.

## 1. Introduction

Dermatophytic pseudomycetoma (DPM) is a rarely reported invasive fungal dermal and/or subcutaneous infection of humans [1] and mammals, especially cats [2,3,4,5,6,7,8,9,10], and occasionally also ferrets [11], dogs [12], and horses [13]. DPM has also been described in extracutaneous locations in a small number of feline cases, including intra-abdominal, intranasal, intraoral, and in cutaneous lymph nodes [2,14,15,16,17,18,19,20]. A breed predisposition for DPM is apparent for Persian cats [2,8,14,15,16,17].

*Microsporum canis* has been identified by macro- and micromorphology of isolates cultured from biopsy tissue in individual cases of feline DPM [7,10,13,16]. Molecular identification has also been performed in a small number of cases, including PCR and sequencing of ITS and 28S ribosomal DNA (rDNA) from fungal culture material in one case or a 183 bp fragment of 18S rDNA extracts from formalin-fixed paraffin-embedded biopsy tissues in seven other cases, all yielding sequences with 100% nucleotide homology to *M. canis* [13,17].

In human medicine, there is a nomenclatural differentiation of “mycetoma” from “pseudomycetoma”. The former can be fungal (eumycetoma) or bacterial (actinomycetoma) in origin and is characterized by the presence of tissue “grains” or “granules” comprised of microcolonies of the infecting bacterium or fungus, as well as draining sinus tracts to the exterior of the involved tissue and traumatic inoculation of an extremity (usually the foot) by the infecting agent [21]. In contrast, pseudomycetomas are characterized histologically by the presence of “pseudogranules” of fungal hyphal microcolonies surrounded by prominent homogeneous eosinophilic material, known as the “Splendore–Hoeppli” reaction [1]. The Splendore–Hoeppli reaction is comprised of antigen–antibody complexes and host inflammatory cell debris [22]. In humans, invasive dermatophyte infections of the deep dermis or subcutis are described as dermatophytic pseudomycetomas [1,23,24] or, confusingly, by the synonymous term “Majocchi’s granuloma” [25,26]. In veterinary medicine, most invasive dermatophyte infections have been described as DPM; however, some cases have been reported as mycetomas [7,9,27].

The aim of this study was to determine the molecular identity of intralesional fungi in histological specimens of DPM in cats and to describe the clinicopathological features of the disease, including the frequency of extracutaneous involvement.

## 2. Material and Methods

### 2.1. Study Design

The laboratory information management system of City University of Hong Kong’s Veterinary Diagnostic Laboratory (VDL) was searched for cases of suspected DPM among feline histopathological submissions from January 2018 to January 2022. Data were extracted from records of identified cases including age, sex, breed, clinical presentation, dermatophyte culture result, histological description, and sequencing results of conventional PCR targeting a 390 bp fragment of the 28S region of the ribosomal DNA gene cluster (28S rDNA) [28]. Veterinarians who had submitted cases with suspected extracutaneous DPM lesions were contacted and invited to contribute medical records from affected patients to provide further information about the clinical presentation.

Blocks of formalin-fixed paraffin-embedded tissue (FFPET) were retrieved from suspected DPM cases for PCR and sequencing of the ITS2 region of the rDNA gene cluster. For cases in which 28S rDNA sequences were not available and ITS2 PCR sequences could not be generated, immunohistochemistry (IHC) to detect dermatophytes was performed.

Cases were included in the final analyses if they had histological findings consistent with DPM, together with confirmation of dermatophyte infection from sequencing of ITS2 or 28S rDNA, or had positive IHC results.

### 2.2. Molecular Identification

Slices of FFPET (5 × 10 µm thickness) were cut from the corresponding tissue blocks of all identified suspected cases of DPM using a sterile microtome blade. DNA extraction was performed using the EZ1 Advanced DNA Paraffin Section Card and Qiagen EZ1 automated DNA extraction robot (Qiagen, Hilden, Germany) according to the manufacturer’s instructions. Before DNA extraction, samples were first deparaffinized in buffer G2 (Qiagen, Germany) at 75 °C for 5 min, digested by addition of proteinase K with overnight incubation at 56 °C, and then boiled for 10 min.

Conventional PCR targeting the internal transcribed spacer 2 (ITS-2) was performed using primers ITS-3-F (5′-GCATCGATGAAGAACGCAGC-3′) and ITS-4-R (5′-TCC TCC GCT TAT TGA TAT GC-3′) [29]. PCR was performed in a 50 μL volume comprising 5 μL of 10X DreamTaq Buffer (Thermo Fisher Scientific, Austin, TX, USA), 5 μL of 2 µM dNTP, 1 μL of each primer (10 µM), 0.25 μL DreamTaq Hot Start DNA Polymerase (Thermo Fisher Scientific, Austin, TX, USA), 35.75 μL molecular-grade water and 2 μL DNA template. The PCR cycling conditions included 1 cycle of 93 °C for 2 min (initial denaturation), 30 cycles of 95 °C for 30 s (denaturation), 55 °C for 30 s (annealing), and 72 °C for 60 s (extension) followed by a final step of 72 °C for 10 min (final extension) [30].

After gel electrophoresis using 1% agarose gel, PCR products were subjected to Sanger sequencing then identified by comparative sequence analysis using the nucleotide–nucleotide Basic Local Alignment Search (BLAST) tool on GenBank (National Institutes of Health). ITS2 sequence results obtained in this study were deposited on GenBank (Accession numbers PP647555 to PP647573 and PP732993).

### 2.3. Immunohistochemistry

In PCR-negative cases, IHC of FFPET incorporating an anti-dermatophyte mouse monoclonal antibody XCMAO1 was performed according to the manufacturer’s instructions (Xceltis GmbH, Mannheim, Germany). This antibody recognizes a fungal cell wall polysaccharide antigen present in dermatophytes, including *Trichophyton species*, *Microsporum canis*, *Nannizzia gypsea* and *Epidermophyton* species [31]. In brief, paraffin sections from cases, along with positive and negative control sections, were cut at a thickness of 4 µm and attached to charged slides (Leica BOND Plus Slides, Leica Microsystems Limited, Hong Kong) before staining with the anti-dermatophyte mouse monoclonal antibody at a 1:100 dilution for 10 min and reviewed by a specialist veterinary pathologist (FH). Tissue sections with the primary antibody replaced by antibody diluent (Leica Biosystems, Newcastle, Australia) served as negative controls. Normal feline skin was used as a positive control.

### 2.4. Statistical Analyses

To indirectly assess the potential association between breed and DPM, the proportions of the breeds of cats in our dataset were compared with the expected proportion of these breeds among all new feline consultations at the hospital affiliated with the VDL, CityU Veterinary Medical Centre (VMC), per year, using the One-Sample Test of Proportion. The annual, expected admission proportions of breeds was estimated by averaging the total number of new consultations in 2018 and 2019. The VMC is the largest veterinary hospital in Hong Kong and receives primary care, specialist, and emergency cases.

## 3. Results

### 3.1. Case Signalment and Anatomic Location of Lesions

In total, 32 samples and data were retrieved from 30 cats with suspected DPM for this study. Two cats had a second sample submitted after disease recurrence. All samples were confirmed as DPM (Table 1).

Of the 30 cats, 19 (63%) were male and 11 (37%) were female. Cats ranged in age from 7 months to 11 years (median 6 years). Affected breeds included 11 Exotic shorthair/longhair (36.7%), 8 British shorthair/longhair (26.3%), 7 Persian (23.3%), and 4 crossbreds (3 domestic shorthair/longhair, 1 British shorthair cross) (13.3%). There were significantly more Persian and Exotic breed cats compared to the expected proportions (3%, *p* < 0.001 and 8.5%, *p* < 0.001, respectively). British-breed cats were not over-represented compared to the expected proportion (21.6%, *p* = 0.817).

In total, 25 (83.3%) cats only had cutaneous lesions, while 5/30 (16.7%) had extracutaneous involvement alone (2 cats) or in addition to cutaneous lesions (3 cats). Concurrent or previous dermatophytosis was noted in 4/30 cats (13.3%) (Table 1).

#### 3.1.1. Cutaneous Lesions

**Figure 1:**
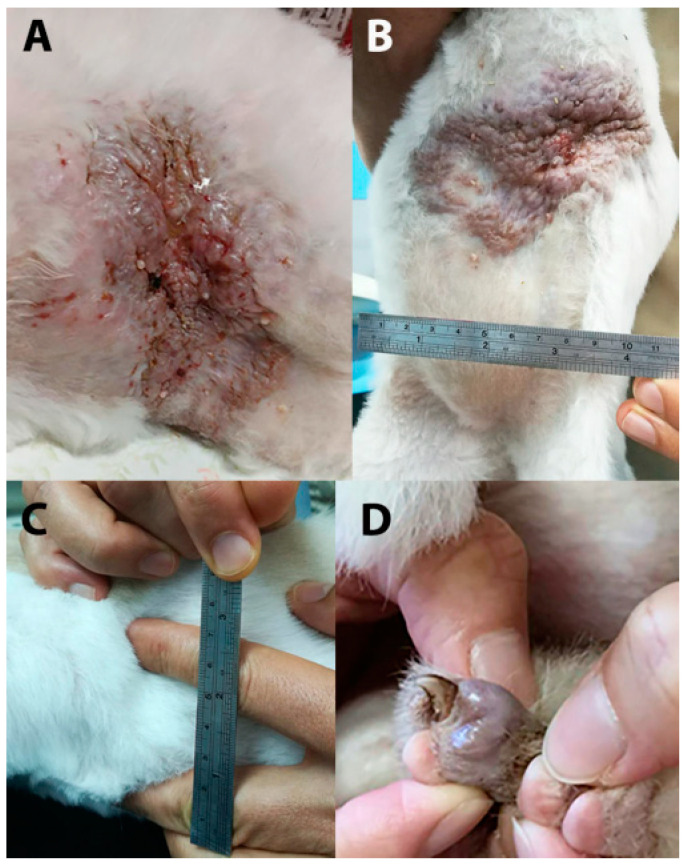
Case 3 (Table 1) was presented with diffuse ulceration and thickening of ventral abdominal skin (**A**). After 3-month treatment with itraconazole then terbinafine, the remaining affected skin (**B**) was resected en bloc, and an enlarged left superficial cervical lymph node (**C**) was biopsied. The cat developed a recurrent focal dermatophytic pseudomycetoma the following year (**D**) involving a hind-limb digit.

There were 28 cats with cutaneous lesions, of which 25 had cutaneous lesions only and 3 also had EC lesions. Of the 25 cats with cutaneous involvement only, age ranged from 0.58 to 11 (median 4) years. Cutaneous lesions were most frequently described as subcutaneous nodules or masses and occasionally as cysts, wounds, or plaques (Figure 1). There were multiple (>2) lesions in 14/27 cats where lesion number was specified (51.9%). Lesions were described as ulcerated in 16/28 cats (57.1%). Anatomic location was described for 31 cutaneous lesions and included the head (3, 9.7%), pinnae (3, 9.7%), neck (2, 6.5%), trunk (15, 48.4%; scapula, lateral thorax, back, flank, axillae, ventral abdomen, mammary gland), limbs (3, 9.7%), digits (3, 9.7%), and tail (2, 6.5%). Lesions were slowly progressive and often described as being present for months before biopsy. DPM lesions were completely excised in 16/24 (66.7%) cases in which excision was attempted (Table 1). Disease-free surgical margins were measured in eight excisional biopsies and all had narrow margins of 0.5–2 mm in at least one dimension.

#### 3.1.2. Extracutaneous Lesions

All cats with extracutaneous lesions were ≥7 years old (5/5), in contrast with 32% (8/25) of cats with cutaneous lesions only. Extracutaneous biopsy tissues with histological evidence of dermatophyte invasion included hard palate, soft palate, superficial cervical lymph node, vagina/cervix (intra-abdominal mass), and retroperitoneal (sub-lumbar mass) in one cat each. Of the five DPM cases with extracutaneous involvement, sequences of *M. canis* were obtained from DNA of FFPET taken from biopsies of the involved extracutaneous tissues in two cases, including hard palate (Case 2) and one of the abdominal masses (Case 8) (Table 1). Medical records were available for review in four of five cases with extracutaneous involvement.

A 7-year-old male neutered Exotic shorthair (ESH) (Case 3, Table 1) had a DPM involving a large area of ulcerated ventral abdominal skin (7 × 12 cm) with concurrent enlargement of the left superficial cervical lymph node (SCLN) (Figure 1). The cat had a history of chronic dermatophytosis. Cutaneous ventral abdominal DPM was first diagnosed in 2016 at another veterinary clinic, with partial response to itraconazole and frequent relapses when medication was stopped. In 2019, the cat presented with diffusely ulcerated and thickened ventral abdominal skin and an enlarged left SCLN. After three months of medical treatment with oral itraconazole followed by terbinafine, en bloc resection of a large remaining plaque-like area was performed. The cat was treated with itraconazole and terbinafine for approximately 3 months and had no further visible lesions. It was represented 9 months later for a subcutaneous digital nodule of the left hind limb, which was biopsied and confirmed to be recurrent DPM (Table 1). Treatment with terbinafine and itraconazole was resumed but on examination 1 year later for vaccination, the lesion had not resolved.

A 7-year-old female spayed ESH cat (Case 20, Table 1) presented for chronic pollakiuria and haematuria of over one-month duration. The cat was found to have a caudal abdominal mass (~4 cm in diameter) associated with the remnant vaginal cervix, that was adhered to the bladder trigone and encompassed both ureters. The mass was incompletely surgically resected, and culture yielded M. canis. The cat was treated with itraconazole 5 mg/kg q 24 h PO. At recheck examination 2 months later, the pollakiuria and haematuria had resolved but the cat was polyuric and polydipsic. Further investigation was declined and a further 2 months of itraconazole was prescribed. The cat did not return for further rechecks.

**Figure 2:**
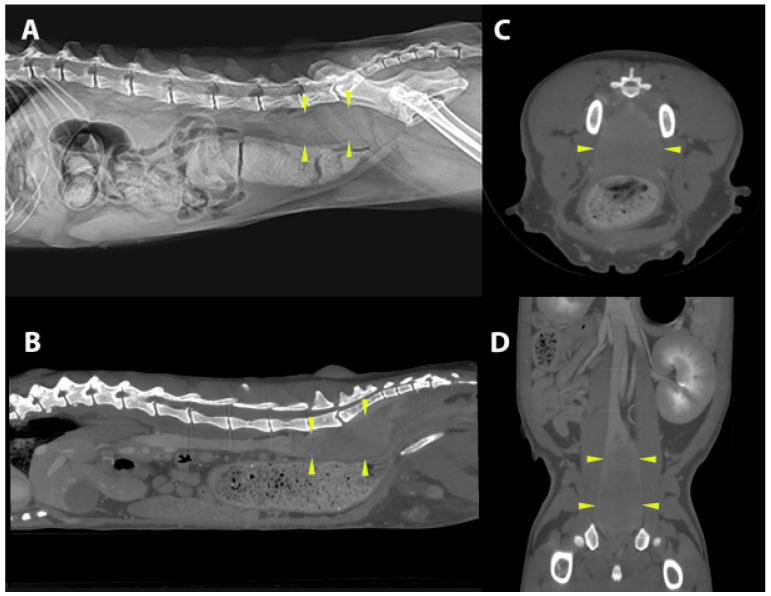
Diagnostic imaging findings in a cat with an intra-abdominal dermatophytic pseudomycetoma and presentation for constipation (Case 8, Table 1). Right lateral radiograph (**A**) and sagittal (**B**), transverse (**C**), and coronal (**D**) computed tomographic images show a soft-tissue density retroperitoneal sub-lumbar mass dorsal to the colon (yellow arrowheads), extending from the cranial border of L6 cranially through the pelvic inlet caudally where it obliterates the rectal lumen. Proximal to the rectum, the colon is distended with faeces.

A 9-year-old male neutered Persian cat (Case 8, Table 1) presented with constipation and a soft tissue mass was palpated in the caudal abdomen. On computed tomography (CT), there was a cylindrical soft tissue retroperitoneal sub-lumbar mass (5 × 2 × 2 cm) dorsal to the colon. The cranial extent of the mass was at the level of lumbar vertebra L6 and caudally the mass extended through the pelvic inlet and attenuated the lumen of the rectum. Proximal to the rectum, the colon was distended with faeces (Figure 2). The mass was surgically debrided and the cat was treated with itraconazole 10 mg/kg q 24 h PO for 6 months. Although constipation resolved initially, the mass gradually increased in size again over time, and recurrent constipation was managed medically. The cat was lost to follow-up 2 years after first presentation, at which time it also had IRIS Stage IV chronic kidney disease.

**Figure 3:**
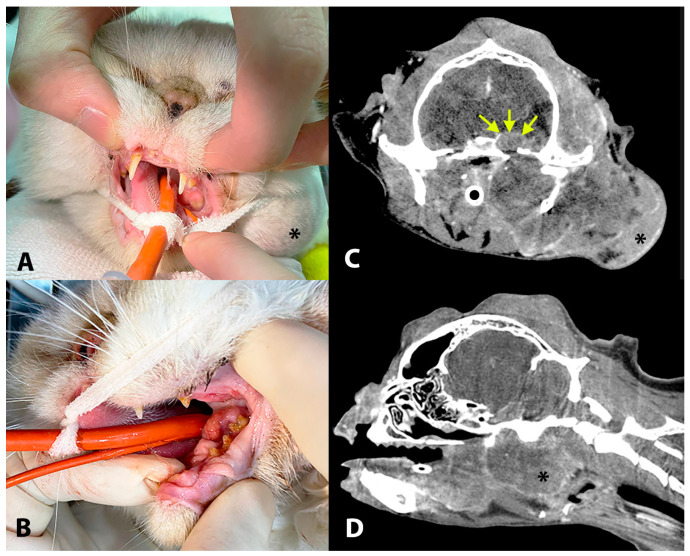
A 10-year-old Exotic shorthair (Case 10, Table 1) with a left-head tilt, stertorous respiration, left retromolar oral mass (**A**,**B**), a large mass over the left submandibular/auricular region (asterisk (**A**,**C**,**D**)), and multiple nodular masses on the dorsal head and neck. A CT scan of the head showed the left-sided facial mass was multilobulated and extended across tissue planes into the oral cavity at the level of the temporomandibular joint, obliterating the caudal nasopharynx (**D**). The mass extended intracranially through a markedly widened oval foramen at the level of the TMJ, showed heterogeneous contrast uptake, and displaced the cerebrum dorsally ((**C**), arrows).

A 10-year-old female spayed ESH (Case 10, Table 1) presented for investigation of inappetence and multiple nodular masses on the head that had first occurred 9 years previously and had been resected but recurred and grew progressively larger. At presentation, the cat had a left-head tilt, stertorous respiration, a soft-tissue mass (2 cm diameter) in the left retromolar region of the oral cavity, a mass over the left submandibular/auricular region (5 cm diameter), and multiple other nodular masses (up to 2 cm diameter) on the dorsal head and neck. A CT scan of the head showed the left-sided facial/aural mass was multilobulated and extended across tissue planes into the oral cavity at the level of the temporomandibular joint (TMJ), obliterating the caudal nasopharynx. The mass also extended intracranially through a markedly widened oval foramen at the level of the TMJ. The intracranial portion of the mass showed heterogeneous contrast uptake and displaced the cerebrum dorsally (Figure 3). An incisional biopsy of the facial/aural mass confirmed DPM (Case 10, Table 1).

### 3.2. Histological, Immunohistochemical, and Molecular Findings

**Figure 4:**
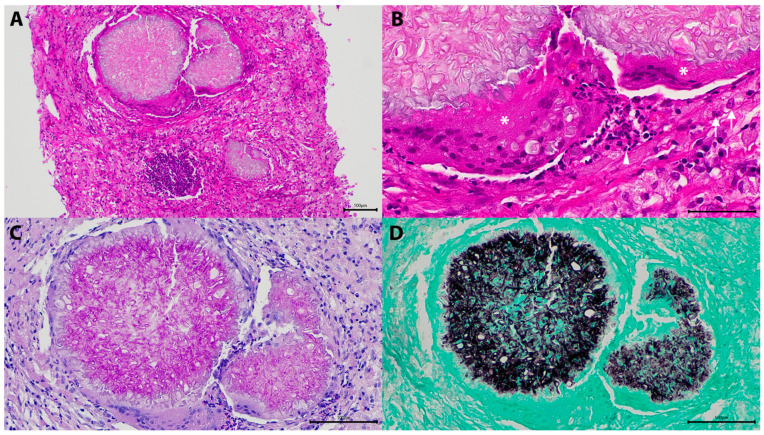
Histological and staining characteristics of a dermatophytic pseudomycetoma in the superficial cervical lymph node in Case 3, Table 1. The lymph node is effaced by a pyogranulomatous cellulitis with multifocal amorphous foci and occasional lymphoid aggregate, hematoxylin and eosin (**A**) (H&E), 100×; (**B**) higher magnification of these amorphous foci reveals matts of fungal hyphae surrounded by epithelioid macrophages (arrow), fewer multinucleated giant cells (asterisks), and neutrophils (arrowhead), H&E, 400×, scale bar = 100 µm.; (**C**) fungal hyphae with bulbous wall highlighted by Periodic Acid Schiff (PAS), 200×; and (**D**) Grocott’s methenamine sliver (GMS), 200×; scale bar = 100 µm.

On hematoxylin and eosin-stained sections, there was a focal to multifocal, granulomatous to pyogranulomatous dermatitis to panniculitis in cutaneous lesions or granulomatous to pyogranulomatous cellulitis in non-cutaneous lesions. Regardless of anatomic location, there were variable infiltrates of epithelioid macrophages admixed with fewer neutrophils, lymphocytes, plasma cells and multinucleated giant cells on a background of fibrous tissue with occasional oedema, haemorrhage, and/or necrosis. Scattered within were multifocal to coalescing aggregates of pink amorphous material with mats of fungal elements (Splendore–Hoeppli reaction), which were non-pigmented, refractile to slightly basophilic, bulbous to parallel 3 to 10 μm, non-septate to septate hyphae, pseudohyphae, with up to 15 μm diameter basophilic conidial/yeast-like elements. Fungal elements stained prominently on special stains (GMS, PAS) (Figure 4).

**Figure 5:**
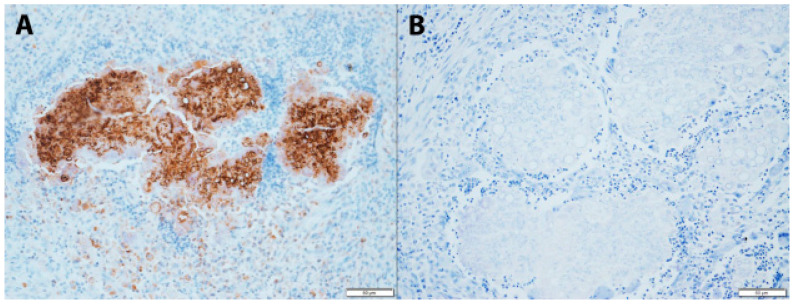
Immunohistochemical staining of a cutaneous dermatophytic pseudomycetoma, Case 1, Table 1, stained with an anti-dermatophyte mouse monoclonal antibody (Xceltis GmbH, Mannheim, Germany). Fungal hyphae in the centre of the lesions exhibit positive (brown) staining (**A**). There is no positive staining of the negative control tissue (**B**). Scale bar = 50 µm.

*Microsporum canis* was identified in 23/24 cases with successful PCR and molecular sequencing results. ITS2 sequences were generated from 22/32 (68.8%) FFPET samples, of which 21 had >99% nucleotide identity to reference sequences of *M. canis* (Genbank accession numbers PP647555 to PP647573 and PP732993). *Trichosporon inkin*, a basidiomycetous yeast, identified from the ITS2 sequence of one case (Case 3, Table 1), was considered a contaminant. A 28S rDNA sequence from a biopsy in the same cat with recurrent disease 1 year later yielded M. canis. One other sample with negative ITS2 sequence results was identified as M. canis from 28S rDNA sequence results. IHC confirmed DPM in all cases for which DNA sequence data could not be generated (Figure 5) (Table 1).

## 4. Discussion

This study of 32 cases of DPM in 30 cats is the largest to be reported thus far since, except for several small case series describing two to eight cases [2,6,8,13], all other reports of feline DPM describe single cases. Our series of 32 DPM cases was amassed over a relatively short time period (4 years) despite the fact that cat ownership in the Hong Kong SAR is relatively low. In 2018, approximately 10% of households reportedly kept dogs or cats and the total number of owned cats was 184,100 [32]. These data, although voluntary and likely an underestimate of the true owned-cat population suggest that DPM may be a more common mycosis in the Hong Kong SAR compared to regions where cat ownership is much higher, but DPM is reported less commonly, such as the United Kingdom [8].

The apparent high prevalence of DPM in Hong Kong may be influenced by feline breed preferences of owners, as well as climactic factors of high heat and humidity. Three breeds of cats predominated in this study—Persians, Exotic short/longhair, and British short/longhair. However, British breed cats are popular in Hong Kong and no significant association with DPM was found based on feline consultation data from the hospital affiliated with the laboratory. These data also suggest that Persian and Exotic breed cats are over represented for DPM. A predisposition for Exotic breed cats for DPM has not been previously reported but is not unexpected since they are closely related to Persian cats. The Exotic breed is derived through the breeding of Persian cats with British shorthair or other shorthair breeds.

A genome-wide association (GWAS) study of 10 Persian cats with severe dermatophytosis (n = 8) or DPM (n = 2) and 16 control cats without dermatophytosis showed affected cats had a highly divergent haplotype for the S100A9 gene, which encodes for a subunit of the antimicrobial peptide calprotectin, with 13/135 differences in amino acids (30). Calprotectin is highly expressed in the skin during dermatophytosis and is thought to have a major role in anti-dermatophyte defense [33]. Similar genetic studies in Exotic breed cats are warranted to determine whether this breed has the same divergent S100A9 gene haplotype as Persian cats.

The only dermatophyte identified in our study was *M. canis*. This is not surprising, since cats are the primary reservoir host of *M. canis* and the pathogenesis of DPM often involves rupture of a dermatophyte-infected hair follicle followed by colonization of the deep dermis or subcutis [9]. Arthrospore contamination of integumentary breaches, together with impaired local immune-defenses such as calprotectin dysfunction, could also enable establishment of DPM. The low rate of cases with concurrent or previous dermatophytosis (13%) reported here was likely underestimated since historical data were not systemically collected. In one report of DPM in four Persian cats, all had multifocal areas of hair-coat fluorescence on Wood’s Lamp examination [2].

Although *Trichophyton mentagrophytes* complex has been described anecdotally to occasionally cause feline DPM, confirmation of an aetiological role is lacking [34]. Causative agents of DPM in humans are anthropophilic dermatophytes such as *Trichophyton tonsurans* and *T. rubrums* and zoophilic species, including *M. canis.* [25].

Cutaneous DPM lesions in our study were most frequently located on the trunk, head and neck, especially dorsally, as has been reported previously [2,8]. However, we also identified lesions on the extremities (digit or pinnae) in five cats, expanding the differential diagnosis list for nodular digital lesions in cats.

Two of the extracutaneous cases in our series study were intra-abdominal and may have resulted from arthrospore contamination of the surgical field during neutering. Intra-abdominal DPM have been reported in three other castrated males [15,16,19] and four female spayed cats [14,16,18,20]. We postulate that retraction of a ligated testicular artery/spermatic cord/tunic contaminated with arthrospores into the caudal abdomen could explain the retroperitoneal sub-lumbar location of DPM in male cats. The anatomical location of the intra-abdominal mass in female cats in our study is similar to previous reports and could have resulted from arthrospore contamination during ovariohysterectomy [14,16].

One cat with extracutaneous lesions in our study had palatine involvement, which has similarly been reported previously in a 6-year-old domestic shorthair cat with dermatophytic fungal rhinitis extending through the hard palate, caused by *M. canis* [17]. The cat in our study also had a cutaneous DPM on the right pinna. Traumatic inoculation of the palate with infected hair/arthrospores during grooming is a possible route of infection in this case.

Another cat in our study with extracutaneous disease had mycotic invasion of a regional lymph node. This finding has been reported in one other cat, which, like our cat, had diffuse plaque-like ulcerated cutaneous lesions [2]. One other cat with extracutaneous disease in our study had an extensive soft-tissue oral cavity mass that extended across multiple tissue planes into the cranium. To the best of the author’s knowledge, this is the first report of intracranial involvement of DPM. Cats with extracutaneous involvement in our study were also older than those with only cutaneous lesions and one cat had a history of cutaneous DPM for >10 years. Intra-abdominal lesions are unlikely to be noticed by owners until their size impacts with organ function to cause gastrointestinal signs (e.g., constipation) or urinary tract signs (e.g., haematuria).

The histopathological findings in cats with cutaneous and extracutaneous DPM in our study fit well within the classification of “pseudomycetoma”. It is also noteworthy that while histopathology was performed in all cases of DPM in this study, biopsy tissue was submitted for fungal culture in only 5/32 cases, suggesting that a fungal aetiology may often not have been considered as a likely differential diagnosis. Definitive identification of the infecting fungal agent using conventional PCR of DNA extracted from FFPET was possible in over two-thirds of our cases, similar to published data in other reports [30,35,36]. False negative results working with FFPET can be caused by cross-linking of DNA with proteins, degradation of DNA during the fixing process, or the presence of PCR inhibitors [37]. Reasons for false positive results on panfungal PCR of FFPET include the presence of commensals in biopsied tissue or contamination of the FFPET block, for example, with environmental fungi [35]. All PCR-negative cases were confirmed as DPM on the basis of positive IHC. The negative control in the commercial IHC kit was comprised of diluent; however, an isotype control is generally superior.

Evidence-based guidelines for treatment of feline DPM do not exist. However, surgical excision alone is not an effective treatment and recurrence at the same or another site is common [2,10]. Amongst the cases here, surgical excision was incomplete in a third of cases and disease-free margins for those that were completely excised were small (<2 mm). Terbinafine or itraconazole, or their combination, has been used to successfully treat cases of DPM [2,4,6,38]. Synergy of treatment with terbinafine and itraconazole has been demonstrated for *Trichophyton* species in vitro and for fungal skin diseases of humans in vivo [39,40]. Antimicrobial susceptibility testing of antifungal drug combinations for *Microsporum canis* isolates is also warranted.

## 5. Conclusions

In this study Persian and Exotic purebred cats were over-represented for DPM, and *M. canis* was the only dermatophyte identified. DPM should be included in the differential diagnoses of cutaneous nodules, masses, or plaques, as well as for slowly progressive EC masses, especially in Persian and Exotic purebred cats. Compared to cutaneous DPM, EC DPM lesions occur in older cats and can have diverse clinical presentations. Although aetiological agents can be identified using PCR of FFPET in approximately two-thirds of cases, clinicians should consider concurrent fungal culture of biopsies from these lesions at the time of submission for histopathology.

## Figures and Tables

**Table 1:** Clinicopathological features of cutaneous and extracutaneous invasive dermatophyte lesions in cats.

Cat no.	Breed	Age-Years	Sex	Lesion Type	Lesion Location	Biopsy	ITS2 PCR	28S rDNA PCR	IHC	Culture	Woods Lamp
1	Persian	7	FN	C, U, S	Neck, dorsal	EB, Co	−ve	ND	+ve	ND	ND
2	Persian	10	Ma	C, U, M EC, S	Right pinna Hard palate	IB	−ve	−ve	+ve	ND	ND
3 *	ESH	7	MN	EC, S	Left SCLN, ventral thorax, abdomen	IB	*Trichosporon inkin*	ND	+ve	ND	ND
3 *	ESH	8	MN	EC, S	Digit, left hind limb	IB	−ve	*M. canis*	+ve	ND	ND
4	DSH	6	F	C, U, M	Interscapular, Limbs	IB	*M. canis*	*M. canis*	ND	ND	+ve
5	ESH	6	F	C, U, M	Trunk (hip to tail)	EB, IC	*M. canis*	ND	ND	ND	ND
6	BLH	2	MN	C, S	Digit, left hind limb (P2)	EB, Co	*M. canis*	ND	+ve	ND	ND
7	BSH	0.58	MN	C, S	Head, dorsal	EB, Co	*M. canis*	ND	ND	ND	ND
8	Persian	9	MN	EC, S	Sub-lumbar mass	IB	−ve	ND	+ve	ND	ND
9	ELH	10	F	C, S	Digit, RHL (P2)	EB, Co	−ve	ND	+ve	ND	ND
10	ESH	10	F	C, M EC, S	Dorsolateral head, Oral cavity, Intracranial	IB	*M. canis*	ND	ND	ND	ND
11 *	BSHX	.8	FN	C, U, M	Dorsal head, trunk, flanks	EB, Co	*M. canis*	ND	ND	−ve	+ve
11 *	BSHX	2	FN	C, S	Pinna, left	EB, Co	*M. canis*	ND	ND	ND	ND
12	BSH	1	MN	C, S	Left hind limb	EB, IC	−ve	ND	+ve	ND	ND
13	ESH	10	MN	C, M	Trunk, ventral abdomen, left lateral thorax	EB, Co	−ve	ND	+ve	ND	ND
14	Persian	1.5	FN	C, U, M	Unknown	EB, IC	*M. canis*	ND	ND	ND	ND
15	ESH	2	MN	C, U, M	Dorsal trunk, interscapular	EB, Co	*M. canis*	ND	ND	ND	ND
16	ESH	7	MN	C, U, M	Trunk, ventral abdomen	EB, Co	*M. canis*	ND	ND	ND	ND
17	BSH	8	MN	C, U, S	Trunk, dorsal	EB, Co	*M. canis*	ND	ND	ND	ND
18	BSH	11	MN	C, U, S	Trunk, ventral abdomen	EB, Co	*M. canis*	ND	ND	−ve	ND
19	Persian	10	MN	C, S	Neck, dorsal	EB, Co	*M. canis*	ND	ND	ND	ND
20	ESH	7	FN	EC, S	Intra-abdominal/vaginal	EB, IC	*M. canis*	*M. canis*	ND	+ve	ND
21	DSH	4	MN	C, S	Trunk, flank	EB, Co	−ve	ND	ND	ND	ND
22 *	BSH	1	FN	C, M	Trunk, dorsal	EB, IC	*M. canis*	ND	ND	ND	ND
23	BSH	10	MN	C, U, M	Trunk, left axilla	EB, IC	−ve	ND	ND	ND	ND
24	Persian	6	MN	C, U, S	Trunk, dorsal	EB, Co	*M. canis*	ND	ND	ND	ND
25	ESH	4	MN	C, U, M	Trunk, dorsal	EB, Co	−ve	*M. canis*	ND	ND	ND
26	ELH	3	Ma	C, U	Unknown	EB, IC	*M. canis*	*M. canis*	ND	+ve	ND
27	DSH	1.5	F	C, U, M	Thorax, bilateral	EB, IC	*M. canis*	ND	ND	ND	ND
28	ESH	5	FN	C, M	Trunk, axilla	EB, Co	*M. canis*	ND	ND	ND	ND
29	Persian	1	MN	C, U, S	Base of tail	EB, IC	*M. canis*	ND	ND	ND	ND
30	BSH	4	MN	C, U, M	Pinna, tail, hind limb	IB	ND	*M. canis*	ND	+ve	+ve

* Previous history of dermatophytosis; +ve, positive; −ve, negative; BSH, British shorthair; BLH, British longhair; BSHX, British shorthair cross; C, cutaneous; Co, complete; DSH, domestic shorthair; EB, excisional biopsy; EC, extracutaneous; ESH, exotic shorthair; F, female; FN, female neutered; IB, incisional biopsy; IHC, immunohistochemistry; Ma, male; MN, male neutered; ND, not done/not reported; M, multiple; S, singular; SCLN, superficial cervical lymph node; U, ulcerated.

## Data Availability

The raw data supporting the conclusions of this article will be made available by the authors on reasonable request.
